# An accurate spectrophotometric method for chitosan quantification

**DOI:** 10.1093/biomethods/bpad036

**Published:** 2023-11-29

**Authors:** Oscar González-Davis, Itandehui Betanzo, Rafael Vazquez-Duhalt

**Affiliations:** Centro de Nanociencias y Nanotecnología, Universidad Nacional Autónoma de México, Baja California, 22860, Mexico; Centro de Nanociencias y Nanotecnología, Universidad Nacional Autónoma de México, Baja California, 22860, Mexico; Centro de Nanociencias y Nanotecnología, Universidad Nacional Autónoma de México, Baja California, 22860, Mexico

**Keywords:** anthrone, carbohydrate, chitosan determination, deamination

## Abstract

Chitosan is a biopolymer obtained from chitin, one of the most abundant biopolymers in nature. Numerous applications of chitosan are well known in the biomedical, environmental, and industrial fields, and the potential applications are considerable. This work reports a new spectrophotometric method to determine chitosan concentration accurately. The method is based on the deamination of chitosan by nitrite in acidic conditions, followed by a carbohydrate determination by the anthrone reagent.

## Introduction

Chitosan is the deacetylated derivative of chitin, one of the most abundant biopolymers on earth. Due to its properties, there is a considerable variety of uses, which include gels for wound treatment [1, 2], drug delivery [3, 4], and bioremediation [5, 6], among others [7].

There are some reported methods for chitosan quantification. The historical colorimetric method was used to quantify chitosan with ninhydrin, a reagent commonly used for recognizing and quantifying amino groups [8]. The reaction was time dependent and showed significant interferences in the presence of proteins and other nitrogenous compounds. Another improved colorimetric method for chitosan determination is using the dye Cibacron brilliant red 3B-A [9]. This method is more sensitive, with better reproducibility than the ninhydrin-based method. In a variation of this method, instead of measuring the spectra of the dispersed chitosan–dye complex, the solution is centrifuged to sediment the colloids, and the concentration of the uncomplexed dye in the supernatant is measured [10]. This simple procedure can improve sensitivity to >2 ppm, and the equilibration time does not influence the measurements. Other anionic dyes, including Acid Orange 9, Acid Red 13, Acid Red 27, Orange II, Alizarin S, Alizarin GG, and Congo Red for colorimetric chitosan determination were reported [9]. Nevertheless, as mentioned above, these methods based on reactions between free amino groups of chitosan and acting agents are unsuitable for the determination of chitosan in the presence of substances bearing primary amino groups, including peptides and proteins [11]. Thus, the chitosan content in protein-loaded nanoparticles could not be determined at all or indirectly estimated without serious errors [12–15].

The derivatization reaction of the primary amino groups of chitosan with *o*-phthalaldehyde and a thiol (*N*-acetyl-l-cysteine) has also been reported [16], as well as method for determination of chitosan in wood and water samples that were developed based on the acidic hydrolysis of chitosan to glucosamine followed by derivatization by *o*-phthalaldehyde, chromatographic separation, and fluorescent detection [17]. High-performance liquid chromatography (HPLC)-based method was reported to quantify chitosan after complete hydrolysis to obtain glucosamine and then derivatized with the chromophore 9-fluorenylmethoxycarbonyl chloride [18]. The hydrolysis of chitosan was optimized using an HCl/H_3_PO_4_ acid mixture.

Finally, a turbidimetric method has been reported [19]. This method is based on the specific reaction between polyiodide anions and chitosan and on measuring the optical density of the insoluble polyiodide–chitosan complex. As an alternative, the content of glucosamine by using capillary electrophoresis has also been developed [20].

Here, an accurate method for determining chitosan concentration is reported. In this method, the chitosan is first deaminated by nitrite in acidic conditions, followed by a carbohydrate determination by the anthrone reagent [21].

## Experimental methods

### Materials and methods

Two chitosan samples reagent grade, one from crab shells (Cat. C-3646, Lot. 98H0504) and another from shrimp shells (Cat. C-3646, Lot. SLCH4865), were obtained from Sigma-Aldrich (St Louis, MO, USA). A food-grade chitosan was purchased from Future Foods S.A. de C.V. (Tlalnepantla de Baz, Mexico). Two chitosan samples from shrimp shells were obtained from the Centro de Investigación en Alimentación y Desarrollo, A.C. (Hermosillo, Mexico). Sodium nitrite, butanol, D-glucose, soluble starch, and glucosamine were obtained from Sigma-Aldrich (St Louis, MO, USA). Acetic acid was purchased from Fermont (Monterrey, Mexico). Anthrone was obtained from Baker Chemicals (J.T. Baker, Phillipsburg, NJ, USA). Ninhydrin solution (0.25% in butanol) was obtained from Pierce Chemicals Co. (Rockford, IL,, USA), and TLC silica gel 60 plates were purchased from Supelco (Merck, Darmstadt, Germany).

### Chitosan deamination

Glucosamine deamination by nitrite has been reported by Bosworth and Scott [22]. The chitosan deamination procedure with some modifications is as follows: 80 mg of NaNO_2_ (1.16 mmol) in increments was added over a 20 min period to 100 mg of chitosan (0.62 mmol) dissolved in 20 mL of 10% acetic acid. The mixture was then heated to 60°C in a capped flask for 24 h to complete the deamination. The absence of amino groups was tested by TLC silica gel 60 aluminum sheets, eluted with 3:1:1 v/v butanol-acetic acid-water, dried, and detected with 0.25% ninhydrin reagent in butanol and heating.

### Anthrone carbohydrate determination

Anthrone reagent was prepared by dissolving 40 mg of anthrone in 20 mL of concentrated sulfuric acid to obtain a 2 mg/mL concentration [21]. This solution was freshly prepared before use. A solution of 10–100 mg/mL of deaminated chitosan was added to a test tube, and the volume was adjusted to 1 mL with distilled water. Then 2 mL of the anthrone reagent was added and mixed and kept on ice until all the samples were heated to 80°C for 15 min. After the samples developed a green-blue color, they were submerged in ice for 5 min and then kept for 5 min at room temperature. Finally, the absorbance was spectrophotometrically determined at 630 nm. Calibration curves were obtained with glucose and starch as standards.

## Results and discussion

Amino carbohydrates such as glucosamine and chitosan cannot accurately be assayed by conventional colorimetric reagents such as phenol sulfuric, anthrone, orcinol, and 2-phenoxyethanol [23, 24]. Because of this, the chitosan should be deaminated to be sensitive to carbohydrate-reactive reagents.

**Figure 1.**
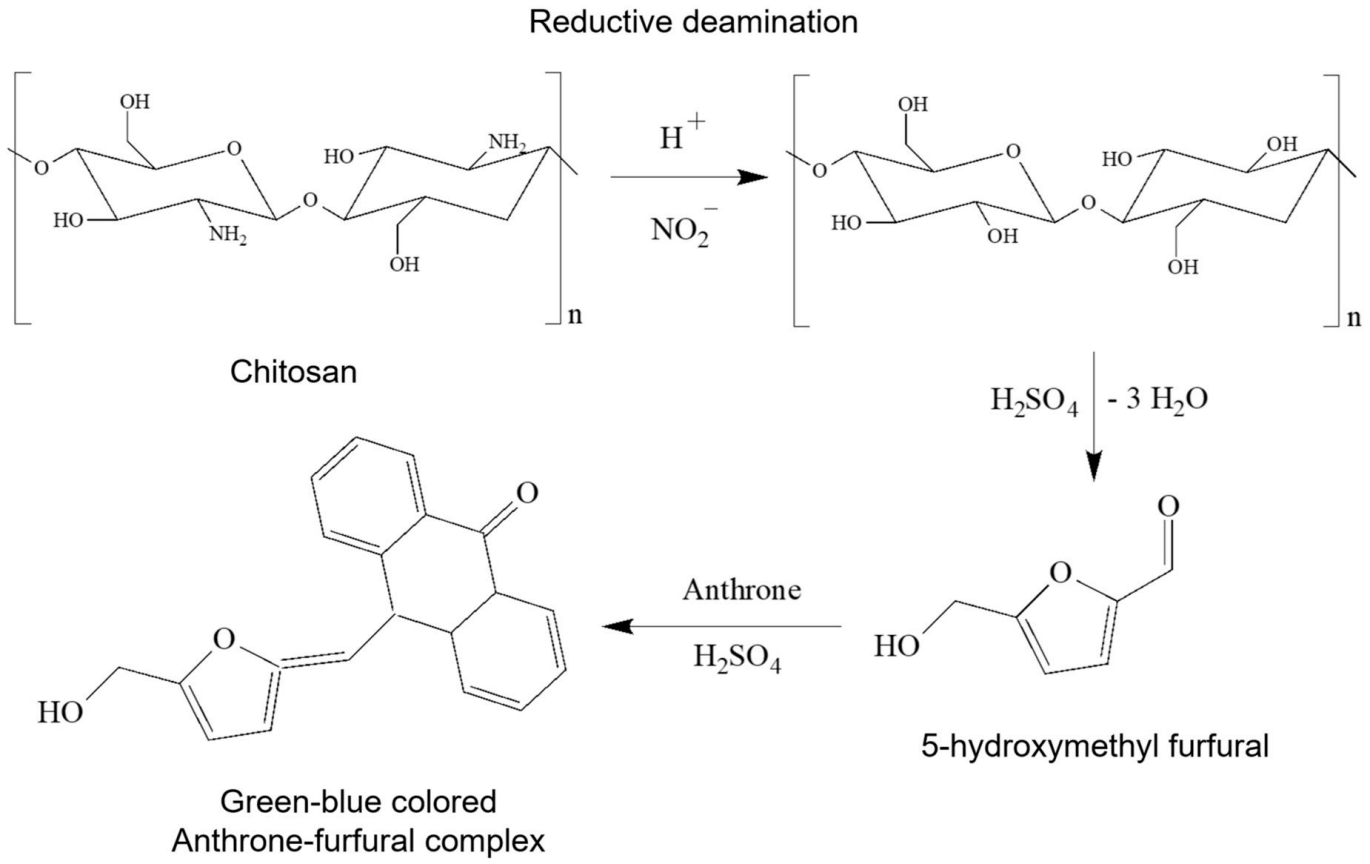
Schematic representation of the chitosan deamination reaction by nitrite in acidic conditions and the colored complexation of furfural derivative with anthrone.

Reactions of sodium nitrite with primary amines cause the replacement of the amino group by a hydroxyl group (Fig. 1). The reaction proceeds rapidly in a weak acidic solution and the presence of a millimolar concentration of nitrite, demonstrating that nitrite induces nitrosative deamination of primary amino compounds. The reaction of nitrous acid with primary amines proceeds through an unstable diazonium intermediate, producing nitrogen and a carbocation (${\text{CH}}_{3}^{⊕}$) unless the diazo group is attached directly to a carbon atom [25, 26]. This reaction occurs by Tiffeneau–Demjanov rearrangement forming 2,5-anhydro-D-mannose from glucosamine [27], and the chitosan deamination proceeds analogously.

In contrast, hexoses and pentoses are hydrolyzed and converted to 5-hydroxymethyl furfural and furfural, respectively, in the presence of sulfuric acid. Furfural then reacts with anthrone to form a color complex showing maximal absorption at 625 nm (Fig. 1) [21].

**Figure 2.**
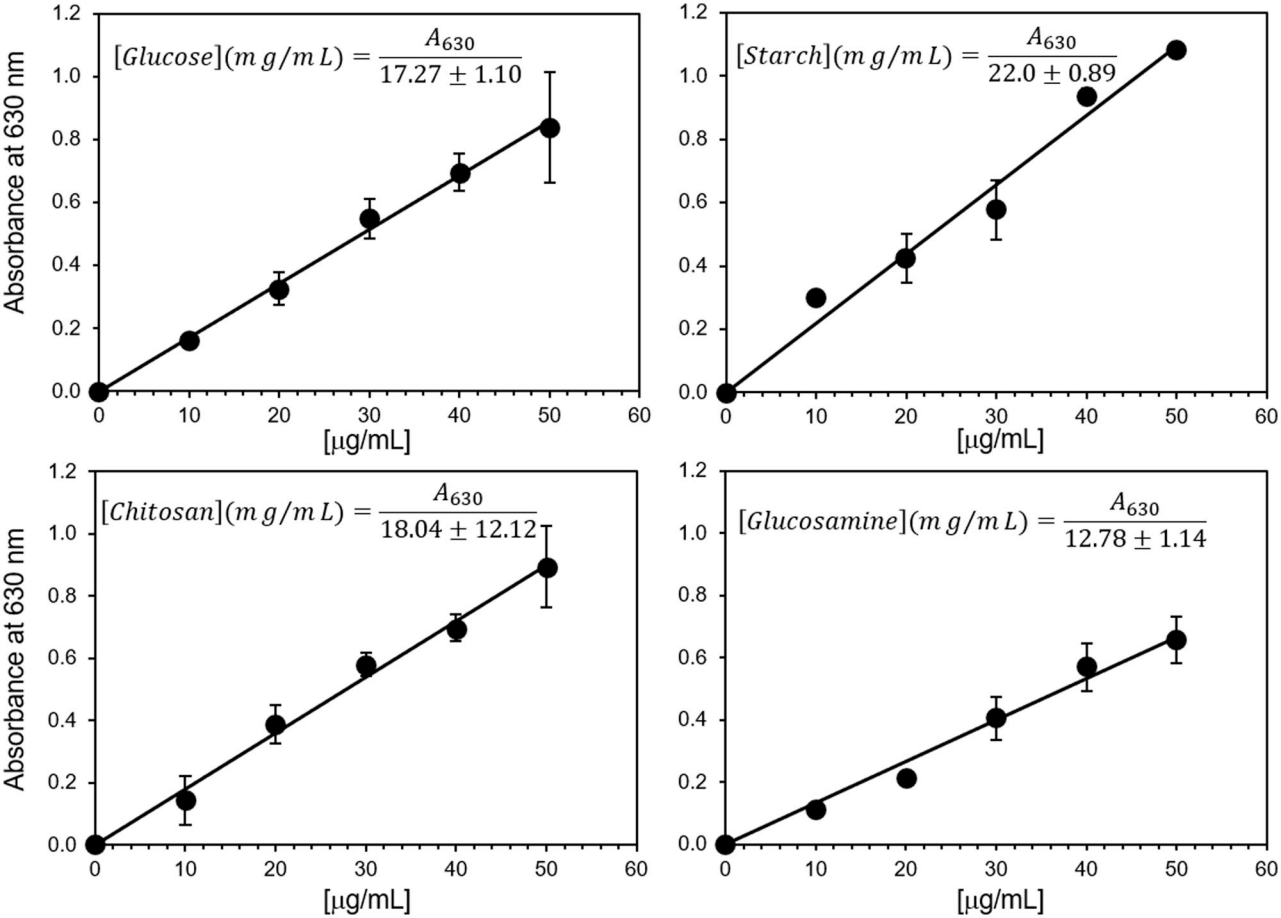
Standard curves of the anthrone reaction obtained with glucose, starch, deaminated glucosamine, and deaminated chitosan. The equations from a linear regression are shown for each standard.

Standard curves were obtained with glucose, starch, deaminated glucosamine, and deaminated chitosan (Fig. 2). All standard compounds showed a linear anthrone response according to the carbohydrate concentration. The proportionality of the response (curve slope) is similar for all the standards except for glucosamine (Fig. 2). The main product of glucosamine deamination is 2,5-anhydro-D-mannose from glucosamine. However, significant amounts of glucuronic acid are also formed [28]. The anthrone reaction with gluconic acid produces a different absorbance spectrum (and therefore color) [24]. The reactivity of the anthrone reagent with the furfural derivative of glucuronic acid shows almost no absorbance at 630 nm and no sharp absorption maximum at 490 nm. Thus, the lower extinction coefficient at 630 nm of the anthrone reaction with deaminated glucosamine could be due to the presence of gluconic acid. The formation of gluconic acid seems to be reduced in the deamination of chitosan due to the presence of the β-(1→4) bond.

Five different chitosan preparations with different molecular weights and deacetylation degrees were quantified (Table 1). The two chitosan preparations from Sigma-Aldrich showed higher purity with chitosan composition close to 100%, indicating the accuracy of our method. In contrast, the chitosan samples prepared in the “Centro de Investigacion en Alimentación y Desarrollo” (CIAD) were shown to contain between 78% and 85% chitosan. Finally, according to our method, the commercial food-grade chitosan contains 76% chitosan.

**Table 1.** Chitosan content determination of different chitosan preparations by the deamination-anthrone method.

Chitosan	Chitosan source	Average MW (Da)	Deacetylation (%)	Sample weight (mg)	Deamination- Anthrone method (mg)	Chitosan content (%)
Sigma-Aldrich	Crab shells	810,000	85	60	63.58 ± 4.10	105.97
Lot. 98H0504						
Sigma-Aldrich	Shrimp shells	585,000	86	60	58.78 ± 1.73	97.97
Lot. SLCH4865						
CIAD-2^a^	Shrimp shells	130,390	92	60	50.97 ± 3.39	84.95
CIAD-1^b^	Shrimp shells	109,500	60	60	46.88 ± 2.00	78.13
Food grade^c^	Future Foods	290,000	90	60	45.42 ± 3.76	75.70

a Sample 2 prepared by the Centro de Investigación en Alimentación y Desarrollo, A.C., Hermosillo, Sonora, México [29].

b Sample 1 Prepared by the Centro de Investigación en Alimentación y Desarrollo, A.C., Hermosillo, Sonora, México [30].

c Future Foods (http://www.futurefoods.com.mx).

The amount of chitosan in the presence of non-amino carbohydrates could be determined by quantifying the carbohydrate concentration before and after deamination and using glucose as standard.


$$\left[\mathrm{Chitosan}\right]\left(µg/ml\right)=\frac{{\left(A_{630}\right)}_{a}-{\left(A_{630}\right)}_{b}}{0.01727}$$

## Conclusions

Amino carbohydrates such as glucosamine and chitosan cannot be quantified by conventional carbohydrate reagents. Nitrite deamination under acidic conditions (HNO_2_ reaction) of chitosan produces 2,5-anhydro-D-mannose that can be determined by its furfural derivative with the anthrone–H_2_SO_4_ reagent. Thus, after deamination, the amount of chitosan can be accurately determined by spectrophotometry.

## Authors’ contributions

Oscar González-Davis (Data curation [equal], Formal analysis [equal], Investigation [equal], Writing—original draft [equal], Writing—review & editing [equal]), Itandehui Betanzo (Formal analysis [equal], Investigation [equal], Methodology [equal]), and Rafael Vazquez-Duhalt (Conceptualization [lead], Data curation [equal], Formal analysis [equal], Funding acquisition [lead], Supervision [lead], Writing—original draft [equal], Writing—review & editing [equal])

*Conflict of interest statement*. None declared.

## Data Availability

Data sharing is not applicable to this article as no datasets were generated or analyzed during the current study.
